# Microbial dynamics in the ripening process of Vorarlberger Alpkäse: farm-specific variations at two Austrian alpine farms

**DOI:** 10.3389/fmicb.2025.1617995

**Published:** 2025-08-13

**Authors:** Johannes Josef Künz, Franz-Ferdinand Roch, Monika Dzieciol, Dmijtri Sofka, Friederike Hilbert, Evelyne Selberherr

**Affiliations:** Clinical Department for Farm Animals and Food System Science, Center for Food Science and Veterinary Public Health, University of Veterinary Medicine Vienna, Vienna, Austria

**Keywords:** cheese ripening, microbial dynamics, alpine pasture, raw milk, microbial community, traditional cheeses, dairy, milk microbiota

## Abstract

**Introduction:**

Vorarlberger Alpkäse (VA) is a Protected Designation of Origin (PDO) Austrian heritage cheese produced from raw milk during the summer grazing period in the Alps. In addition to conducting clinical examinations of cows whose raw milk is used for VA cheese production, this study investigates the microbiota of raw milk and cheese from two alpine farms.

**Methods:**

Raw milk, curd, and cheese core samples were collected and analysed using qPCR and 16S rRNA gene amplicon sequencing to determine bacterial loads and the microbial community composition.

**Results:**

The microbial community in raw milk showed significant variation in alpha diversity and taxonomy during the alpine sojourn, with *Pseudomonas*, *Staphylococcus, Paenibacillus* and *Lactobacillus* being dominant. The cheese curd microbiota was initially diverse, with *Pseudomonas, Acinetobacter*, the *Shigella* complex, *Lactobacillus* and *Hafnia-Obesumbacterium* being most abundant. During ripening, *Lactobacillus* became the predominant genus, while the overall diversity decreased.

**Discussion:**

Differences in microbial communities were observed between the two farms, likely due to variations in production conditions and domestic microbiota. These findings highlight the impact of alpine farming practices on the raw milk and cheese microbiota, influencing the unique characteristics of VA.

## Introduction

Fermented dairy products have long been integral to the economies and cultures of various geographical regions (Wiley, 2007). The Austrian Vorarlberger Alpkäse (VA) is an artisanal surface washed hard cheese made of raw milk from Austria’s westernmost federal state, Vorarlberg. VA has a Protected Designation of Origin (PDO), according to the Council Regulation of the European Union No. 2024/1143 (Regulation (EU) 2024/1143 of the European Parliament and of the Council, 2024). VA is produced following the PDO-AT-1413 protocol, which includes as key criteria, that (i) the alpine pastures must be located at altitudes of 1,000 meters to 1,800 meters, (ii) only fresh raw milk and no tank milk is used, (iii) the feed may only consist of alpine grasses, and (iv) feeding with silage is strictly prohibited. The supplementary feeding of grain meal from other regions is permitted up to an extent of 20% of the dry matter on an annual basis. As a result of this regulation, VA can only be produced in the summer months (May–September), limiting its production quantity. Beyond its gastronomic and economic significance, VA plays a crucial role in maintaining Vorarlberg’s mountain agriculture. Cheese production is essential for the ecological diversity and stability of the region’s alpine cultural landscape. The exceptional quality and extended shelf-life of VA result from the high-quality milk, which is influenced by the alpine microbiome, climate, and traditional artisanal production techniques. Small-scale alpine dairy farms have preserved these methods over generations, ensuring that the cheese maintains its distinctive characteristics. The alpine vegetation and predominantly green diet of the animals is supposed to impart flavor to the milk, which, combined with meticulous artisanal techniques and careful maturation, defines the properties of VA (Regulation (EC) 1107/96 eAmbrosia Union register. Vorarlberger Alpkäse, n.d.; Regulation (EC) 1107/96 eAmbrosia Union register. Vorarlberger Bergkäse, n.d.).

Importantly, traditional Alpine cheese production is deeply intertwined with cultural heritage and identity, as evidenced by archaeological findings indicating milk processing at high altitudes in the Swiss Alps over 3,000 years ago (Reitmaier and Möckli, 2022). Contemporary efforts to protect and promote Alpine cheeses through quality schemes, geographical indications, and local branding initiatives aim to balance cultural preservation with evolving consumer demands (Delfosse, 1998; Grasseni, 2011; Ledinek Lozej, 2021).

Not only in VA but in all raw milk cheeses, raw milk serves as a foundational source of microbial diversity, which significantly shapes the production, maturation, and overall quality of cheeses (Montel et al., 2014). This microbial diversity is not static; rather, it evolves over time and is continuously influenced by factors including the initial raw milk microbiota, processing conditions, environmental fluctuations, and ripening methods (Irlinger et al., 2024; Melkonian et al., 2023; Montel et al., 2014; Tilocca et al., 2020). During cheese production, both starter lactic acid bacteria (SLAB) and non-starter lactic acid bacteria (NSLAB) can be used and play essential roles, with SLAB primarily initiating the fermentation process and NSLAB contributing to flavor development and maturation as ripening progresses (Beresford et al., 2001; Gatti et al., 2014; Neviani et al., 2013; Secchi et al., 2023; Somers et al., 2001). It is known that the microbial diversity of raw milk cheese is influenced by the natural milk culture derived from alpine pastures, which enhances beneficial bacteria such as *Lactococcus*, *Bifidobacterium*, and *Lactobacillus* while reducing undesirable genera like *Pseudomonas* [Alpine pasture microbiota; (Carafa et al., 2020)].

Moreover, *Propionibacterium* present in Swiss raw milk from lowland and alpine regions impacts the organoleptic properties and safety of the resulting cheeses, highlighting the influence of regional factors on microbial community structure, which has some similar characteristics as VA (Fessler et al., 1999). Studies have demonstrated that LAB such as *Streptococcus thermophilus* and *Enterococcus* spp. are prevalent in raw milk used for Fontina cheese production, contributing to flavor development and microbial safety during ripening (Giannino et al., 2009). Furthermore, fungal communities on cheese rinds, predominantly composed of *Mucor* and *Penicillium* species, are shaped by environmental conditions such as temperature, humidity, and cheese type, underscoring their pivotal role in surface-ripened cheeses (de Respinis et al., 2023). These complex interactions between microbial communities and environmental conditions are further reflected in the technological properties of milk, where alpine hay and high-altitude conditions are known to reduce milk protein content and impair cheesemaking properties compared to lowland hay (Leiber et al., 2005). Additionally, the botanical composition of alpine pastures plays a crucial role in shaping the microbial community and cheese composition, with distinct microbiota profiles emerging from cows grazed on *Agrostis-Festuca* dominated pastures (Povolo et al., 2013).

Currently, there are no data available on the microbial community of VA, however research exists for Vorarlberger Bergkäse (VB), which is produced year-round in dairies located in the valleys of the same region. VB is made similarly from cow raw milk that is primarily fed hay. It has been shown that LAB, including *Lactobacillus*, *Lactococcus*, and *Streptococcus* species, initiate fermentation (often they are also used as starter cultures) by rapidly acidifying the curd and providing a suitable environment for ripening-associated microbes (Schornsteiner et al., 2014). On the cheese rind, a complex microbial community dominate the later stages of ripening, contributing to proteolysis, lipolysis, and the development of complex flavor profiles through the production of volatile organic compounds (Quijada et al., 2022). *Brevibacterium, Corynebacterium, Halomonas* and *Staphylococcus* species are the most abundant and metabolically active genera in the cheese rind during VB maturation, and metabolic process results in the characteristic rind formation and sensory attributes of VB (Quijada et al., 2018; Quijada et al., 2020; Quijada et al., 2022; Schornsteiner et al., 2014). Previous studies on the rind of VB cheese have also shown that the microbial diversity increases during the ripening process, whereas the diversity in the cheese core is stable (Quijada et al., 2020; Schornsteiner et al., 2014). The rind microbial community is subjected to significantly different environmental conditions than the microbiota in the cheese core (Almena-Aliste and Mietton, 2014).

The aim of this study was to follow the maturation process of VA produced on two alpine farms and to determine its microbial diversity. It was hypothesized that the microbial diversity of VA cheese would differ between the two Alpine farms due to variations in production conditions and the influence of the domestic microbiome. This research aims to provide insights into how natural microbiota and environmental factors shape the microbial diversity in artisanal cheese production.

## Materials and methods

### Facilities

**Figure 1:**
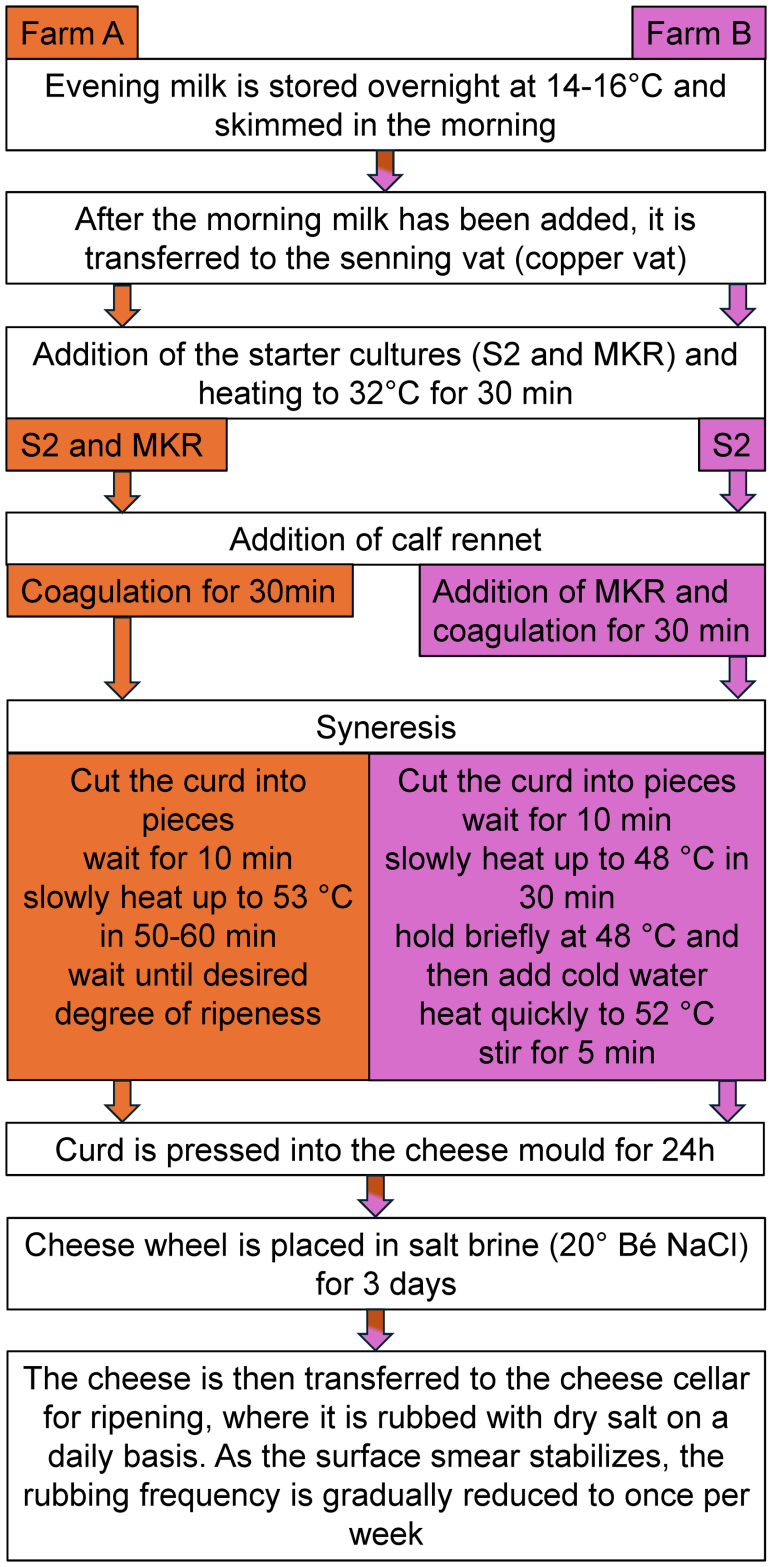
Production process of cheese in farm A and farm B. The flowchart illustrates the cheese-making process at two different facilities, **(A)** left, orange and **(B)** (right, purple), with differences in starter culture usage, coagulation, heating, and syneresis procedures. Bé, Baumé scale (liquid density).

Two alpine farms were selected for this study, both producing their own VA cheese using slightly different production technologies. Farm A is a traditional alpine hut with a tethered for 27 cows, using a tube milking system and producing cheese without modern equipment. Farm B is a newly renovated alpine hut with a new loose housing system for 67 cows, equipped with a milking parlor and a dairy room with modern equipment including a curd pump. At farm A, the teats were wiped dry with wood wool, whereas at farm B, cleaning was done with moist disposable teat cleaning wipes. Both farms produce the VA according to the established production protocol (Figure 1).

### Animals

All cows underwent a standardized general clinical examination, which included assessments of general behavior, breed, posture, nutritional status, internal body temperature, auscultation of heart, lungs, abdomen and rumen activity, palpation of the udder and an examination of feces according to Baumgartner (2014). This examination was carried out at three time points during the alpine season: shortly before moving to the alpine pasture (P1), 1 week after arrival on the alpine pasture (P2), and in the mid-season (P3). Values within the physiological range were noted as “o.B.” (no abnormalities) and deviations were recorded. Some abnormities, such as skin lesions, did not automatically indicate a systemic disease. Additionally, information on name, breed, age, previous illnesses, and antibiotic treatment in the last 6 months was recorded. The milk sampling took place after the farmer had cleaned the udder teats as usual before milking. A bulk milk sample (50 mL) was collected from all four quarters of six randomly selected cows per farm. In addition, from all cows a sterile milk sample was collected from each udder quarter. This procedure was done as described in Baumgartner (2014). The teat ends (teat canal openings) were cleaned twice using alcohol-soaked cotton swabs in a circular motion. Sample collection was done by (1) loosening the sterile cap of the sampling tube (plastic or screw cap) using both hands without removing it, (2) turning the tube so the cap faces down (pronation of the left hand), (3) using the right hand to hold the teat, and milking directly into the open sampling tube, held horizontally. Additionally, conventional bacteriological examination (described in section raw milk samples) was performed. The six randomly selected cows had an additional sampling at the end of the alpine season (P4). On farm A, a cow died in the summer, therefore samples P3 and P4 could not be obtained. All statistical calculations on clinical examination were carried out in Excel (Microsoft Corporation, 2018).

### Raw milk samples

A conventional bacteriological test was carried out on the sterile raw milk samples collected from all cows. The samples (82 g) were centrifuged at room temperature for 15 min, the supernatant was discarded, and the pellet was streak on a Columbia agar + 5% sheep blood (bioMérieux, Vienna, Austria) using a sterile loop. After 24 h of incubation at 37°C, the colonies were identified based on their biochemical profile by VITEK® 2 Compact (bioMérieux, Vienna, Austria). Subsequently, these bacteria were further tested for antimicrobial resistance using microdilution by VITEK^®^ 2 AST (antibiotic susceptibility testing, bioMérieux, Vienna, Austria) cards. The bulk milk samples collected from the 12 cows were centrifuged for 30 min at 3,000 × g. The supernatant was discarded, the pellet was resuspended in 1 mL 1 × PBS (A0965; AppliChem; Darmstadt, Germany) and washed twice in 1 × PBS. The pellet was frozen at −30°C until further use.

### Vorarlberger Alpkäse production

Cheese samples were obtained from both farms (farms A and B), where raw milk was processed directly at the farm as described in Figure 1. The production is based on the use of raw milk and the addition of starter cultures. The starter cultures on both farms contained: S2 (multiple different strains of *Streptococcus thermophilus*) and MKR [mix culture of *Streptococcus thermophilus* (85–90%) and *Lactobacillus delbrueckii* subsp*. lactis* (10–15%)] (HBLFA Tirol, 2024).

VA is treated either in a salt bath or with dry salting of the surface. The first stage of the ripening period takes place in the Alps and is later transferred to the valleys to the same specific ripening facility in Vorarlberg for further maturation. Depending on the space from the cheese ripening cellars, the initial period of ripening in the Alps can last up to 2 months. The traditional aging period is up to 18 months, although this type of cheese can also be sold earlier at three, six and nine months of ripening.

### Curd and cheese core samples

The samples were processed according to the MASTER protocol (Barcenilla et al., 2024). To examine the curd communities, a sample of the curd was taken from each farm during production after the addition of starter cultures and before pressing into the cheese moulds. Curd samples (10 g) were vortexed and homogenized with 2 mL 1 × PBS, then centrifuged for 30 min at 3,000 × g. The supernatant was discarded, the pellet was resuspended in 1 mL 1 × PBS and centrifuged for 15 min at 3,000 × g, washed twice in 1 × PBS and the pellet was then frozen at −30°C until further use.

To investigate the core microbiota, six wheels of cheese (each about 30 kg) from each farm were sampled at three time points during the ripening process. After three (R1), six (R2), and nine (R3) months maturation, samples (~10 g) were taken from the cheese core using sterile cheese trier. Each sample was diluted 1:10 (w/v) with 1 × PBS and mixed at 260 rpm for 2 min in a laboratory homogenizer (Stomacher 3,500 Seward, UK). The homogenate was divided equally into two 50 mL tubes and centrifuged at 15,000 × g for 15 min. The supernatant was poured off. The pellet was then washed twice with 40 mL 1 × PBS. For a last step, the pellet was resuspended in 1 × PBS and then centrifuged at 15,000 × g for 15 min. The pellet was frozen at −20°C until further use.

### DNA extraction

DNA extraction for raw milk and curd samples was performed using the QIAamp Fast DNA Stool Mini Kit (Qiagen, Hilden, Germany) following the manufacturer’s instructions. DNA extraction for the cheese samples was performed according to the MASTER protocol (Barcenilla et al., 2024) and by using the DNeasy PowerSoilPro (Qiagen, Hilden, Germany) as DNA extraction kit. DNA extracted from cheese was eluted twice with 25 μL of 70°C prewarmed diethylpyrocarbonate (DEPC)-treated water, instead of using the C6 solution (Barcenilla et al., 2024). DNA concentrations were determined using the Qubit® ds HS and BR Assay Kit (ThermoFisher Scientific, Vienna, Austria).

### DNA quantification

DNA (1 μL) from all VA samples was pooled, and a conventional PCR was performed using the primer set 341F (5′-CCT ACG GGA GGC AGC AG-3′) and 534R (5′-ATT ACC GCG GCT GCT GG-3′) (Muyzer et al., 1993) to generate the required amount of DNA for the qPCR standard. The resulting DNA was purified using the innuPREP PCRpure Kit (IST Innuscreen GmbH, Germany) and quantified with the Qubit™ dsDNA BR Assay Kit (Invitrogen™). To determine the copy numbers of bacterial 16S rRNA genes, the purified DNA was 10-fold diluted and tested in duplicate in a 20 μL reaction mixture with 9.7 μL of DEPC-treated water, with the final concentrations for the reaction: 10 × buffer 1 (Invitrogen; Carlsbad, USA), MgCl_2_ 2 mM (stock concentration 50 mM, Invitrogen; Carlsbad, USA of each primer), 2.5 μM (stock concentration 2.5 μM, Microsynth, Vienna, Austria), 0.5 μL of EvaGreen^®^ fluorescent DNA stain (JenaBioscience, Jena, Germany), dNTP Mix 0.2 μM (stock concentration 20 mM, 5 mM of each dNTP; Thermofisher, Vienna, Austria), Platinum Taq DNA polymerase 1.5 U (5 U μl^−1^; Thermo Fisher Scientific, Vienna, Austria), and 5 μL of the template (gDNA). Quantification of DNA was performed in a Mx3000P qPCR instrument (v.4.10; Stratagene, La Jolla, CA, United States) with an initial denaturation at 95°C for 3 min, followed by 45 cycles of 95°C for 5 s, 60°C for 20 s. Final copy numbers were calculated in Excel (Microsoft Corporation, 2018) by multiplying the mean of the copy number of extracted DNA (bacterial cell equivalents, BCE) per sample, as determined by qPCR, in 50 μL of cheese samples and in 200 μL of milk samples, (100-fold diluted).

The 16S rRNA gene qPCR data was expressed as bacterial cell equivalents, BCE per 1 mL milk or 1 g cheese core and was analyzed and compared using built-in R stats (version 4.4.2). Data were categorized into milk (M) and cheese samples (C) from two farms (A, B), considering different sampling time points for milk (alpine sojourn phases: P1, P2, P3, P4) and ripening stages for cheese (R1, R2, R3). The Shapiro–Wilk tests indicated that only a subset of the data followed a normal distribution (Supplementary Tables 1, 2). Therefore, all data subsets were summarized using median and interquartile range (IQR). Statistical differences between the same time points (milk: P1-P4; cheese core: R1-R3) across two farms were calculated using the Mann–Whitney U test. Statistical significance was set at *α* = 0.05.

### Sequencing

The construction and sequencing of 16S rRNA gene amplicon libraries were conducted at Procomcure Biotech GmbH, Breitwies, Austria. Library preparation followed the Illumina 16S rRNA Gene Amplicon Sequencing Library Preparation guidelines, targeting the V3/V4 region of the 16S rRNA gene. Amplification was carried out using primers 341F (5’-CCT ACG GGN GGC WGC AG-3′) and 805R (5’-GAC TAC HVG GGT ATC TAA TCC-3′) (Klindworth et al., 2013), in combination with Illumina adapter sequences (5’-CGT CGG CAG CGT CAG ATG TGT ATA AGA GAC AG-3′ and 5’-GTC TCG TGG GCT CGG AGA TGT GTA TAA GAG ACA G-3′). The Nextera XT DNA Library Preparation Kit (Illumina, San Diego, USA) was used for library construction, where sequencing adapters and indices were ligated to the purified PCR products. Equimolar concentrations of amplicons were pooled and sequenced on an Illumina MiSeq platform using a 300 bp paired-end read protocol. This approach resulted in a median sequencing depth of 107,122 reads per sample.

### Bioinformatic analysis

The DADA2 plugin (Callahan et al., 2016) in QIIME 2 (2023.9) (Bolyen et al., 2019) was used for sequence quality control, including denoising and chimera removal. Sequences were truncated at 270 for forward reads and 230 for reverse reads. Taxonomic classification was performed using a pre-trained Naive Bayes classifier, with sequences classified against DAIRYdb, a curated reference database for improved taxonomy annotation of 16S rRNA gene sequences from dairy products (Meola et al., 2019). Results were used to filter sequences, except those identified as chloroplasts, mitochondria, eukaryotes, and unassigned reads. Instead of clustering sequences into operational taxonomic units (OTUs) based on arbitrary similarity thresholds, DADA2 infers Amplicon Sequence Variants (ASVs)-exact biological sequences differing by as little as one nucleotide (Callahan et al., 2016).

The processed data were imported into R (v4.1.2) (R Core Team, 2021) for further statistical testing and visualization. The package “phyloseq” (v1.42.0) (McMurdie and Holmes, 2013) was used to work with the sequencing data in R. To estimate alpha diversities, we calculated Hill-Simpson and Hill-Shannon indices using the “iNEXT” (v3.0.0) (Hsieh et al., 2016) package, based on 99% coverage on diluted samples. Comparisons of alpha diversity were conducted with Kruskal-Wallis tests followed by Bonferroni-alpha-corrected Dunn’s tests for pairwise comparisons. For the Kruskal-Wallis test, a *p*-value of 0.05 was considered significant, and for Dunn’s test, a p-value of 0.025 was used. Beta diversity was assessed using Bray–Curtis dissimilarities calculated from the ASV abundance table. To evaluate the assumption of homogeneity of group dispersions, we first applied the betadisper function from the vegan R (v. 2.6–6.1) package. To test for significant differences in community composition between groups, a PERMANOVA (Permutational Multivariate Analysis of Variance) was performed using the adonis2 function from the vegan R package. The analysis was based on 999 permutations (Oksanen et al., 2022).

## Results

### Clinical examination of animals

The clinical examination data of all cows can be found in the Supplementary Table 3, which provides an overview of the clinical findings. Data of the 12 cows followed over the alpine pasture can be seen in Table 1; one cow (farm A) showed a slightly increased respiratory noise during the first examination. At the second time point, a cow (farm B) showed symptoms of reduced feed intake, dullness, and acetone-like breath suggesting a ketosis. Unfortunately, one cow from farm A died between the second and third examination due to an accident. All remaining cows did not show any clinical symptoms at any time point. The herd size changed slightly during the study on the alpine pasture. On farm A, a cow that had sustained injuries in an accident, was euthanized. On farm B, the number increased by two cows, because they were included in milk production after calving. A summary of the clinical and bacteriological examination findings for both farms is presented in Table 2. The results of individual cows from herds of both farms can be found in Supplementary Table 3.

**Table 1:** Clinical examination of the 12 cows included in this study at three time points during the alpine season: shortly before moving to the alpine pasture (P1), 1 week after arrival on the alpine pasture (P2), and in the mid-season (P3).

Time point	Farm	Cow	Body temperature in °C	Clinical examination	Conventional bacteriological examination of milk for pathogens
P1	A	6	38.1	o.B.	neg.
		10	38.6	Lungs: slightly increased respiratory noise on both sides	neg.
		17	38.0	o.B.	neg.
		19	38.2	o.B.	neg.
		22	38.0	o.B.	neg.
		25	38.3	o.B.	neg.
	B	18	38.3	o.B.	neg.
		36	38.9	o.B.	neg.
		41	38.0	o.B.	neg.
		48	38.1	o.B.	neg.
		51	38.3	o.B.	neg.
		67	38.3	o.B.	neg.
P2	A	6	38.0	o.B.	neg.
		10	38.0	o.B.	neg.
		17	37.7	o.B.	neg.
		19	38	o.B.	neg.
		22	38.3	o.B.	neg.
		25	38.2	o.B.	neg.
	B	18	38.2	o.B.	neg.
		36	39.1	Ketosis	LR *S. warneri*
		41	38.3	o.B.	neg.
		48	36.6	o.B.	neg.
		51	38.4	o.B.	neg.
		67	38.6	o.B.	neg.
P3	A	6	38.5	o.B.	neg.
		10	38.6	o.B.	neg.
		17	38.3	o.B.	neg.
		19	38.6	o.B.	neg.
		22	euthanized		
		25	38.9	o.B.	LF + LR *S. viridans* group except *S. pneumoniae*
	B	18	38.3	o.B.	neg.
		36	38.3	o.B.	neg.
		41	38.4	o.B.	neg.
		48	38.2	o.B.	neg.
		51	38.5	o.B.	neg.
		67	38.7	o.B.	neg.

o.B., without clinical findings; RR, Right rear; LF, Left front; LR, Left rear; neg., negative.

**Table 2:** Overview of the clinical and bacteriological examination at three time points during the alpine season: shortly before moving to the alpine pasture (P1), 1 week after arrival on the alpine pasture (P2), and in the mid-season (P3).

Examination point	P1		P2		P3	
Farm	A	B	A	B	A	B
Date (dd/mm/yyyy)	09/05/2022	31/05/2022	22/05/2022	06/06/2022	08/08/2022	09/08/2022
Number of cows	27	67	27	68	26	69
Clinically notable	4	3	3	5	0	5
Bacteriological screening of milk samples	7	29	6	42	11	19

### Bacteriological examination of cow milk samples

The conventional bacteriological tests of the milk samples from the 12 cows at the time point P1 were all negative for pathogens. At P2, the milk from a cow on farm A tested positive for *Staphylococcus warneri*. At P3, a milk sample from a cow on farm A tested positive for *Streptococcus viridans* group, except *S. pneumoniae* (Table 1). Detailed results of the bacteriological analyses are presented in Supplementary Table 4.

### Quantification of bacteria in raw milk, curd and cores throughout ripening using a 16S rRNA gene qPCR assay

To determine the bacterial absolute abundance in milk samples collected at different alpine sojourn times (P1-P4), in cheese curds, and cheese core samples (R1-R3) from two farms (A, B), qPCR was performed. Overall, BCE/g values in cheese core samples were comparable between the farms (median/IQR, farm A: 2.01e+08/2.68e+08; farm B: 3.44e+08/4.65e+08) (Supplementary Figure 1; Supplementary Table 1). Interestingly, milk samples from farm B showed a significant higher bacterial 16S rRNA gene copy number than those from farm A (median/IQR: A: 3.82e+02/1.51e+03; B: 8.13e+03/2.31e+04, *p* < 0.01), particularly during the early alpine stage (P2, *p* < 0.05, Supplementary Table 2). This suggests farm-specific variation in BCEs in the raw cow milk used for Alpkäse cheese production. BCE/g values in fresh cheese curd were lower to those in cheese samples (A: 8.62e+03; B: 3.38e+04). No statistically significant differences in BCE/ml between farms were observed for milk samples collected during P1, P3, and P4 (Supplementary Table 5). In cheese core samples, also no statistically significant differences in BCE/g core were found across ripening time points (3, 6, 9 months) (Supplementary Table 5). Interestingly, the BCE/g values were highest at the first cheese core sampling time points (3 months) and remained relatively stable in both farms (Supplementary Figure 1) during the ripening time.

### Genomic analysis of raw milk samples

**Figure 2:**
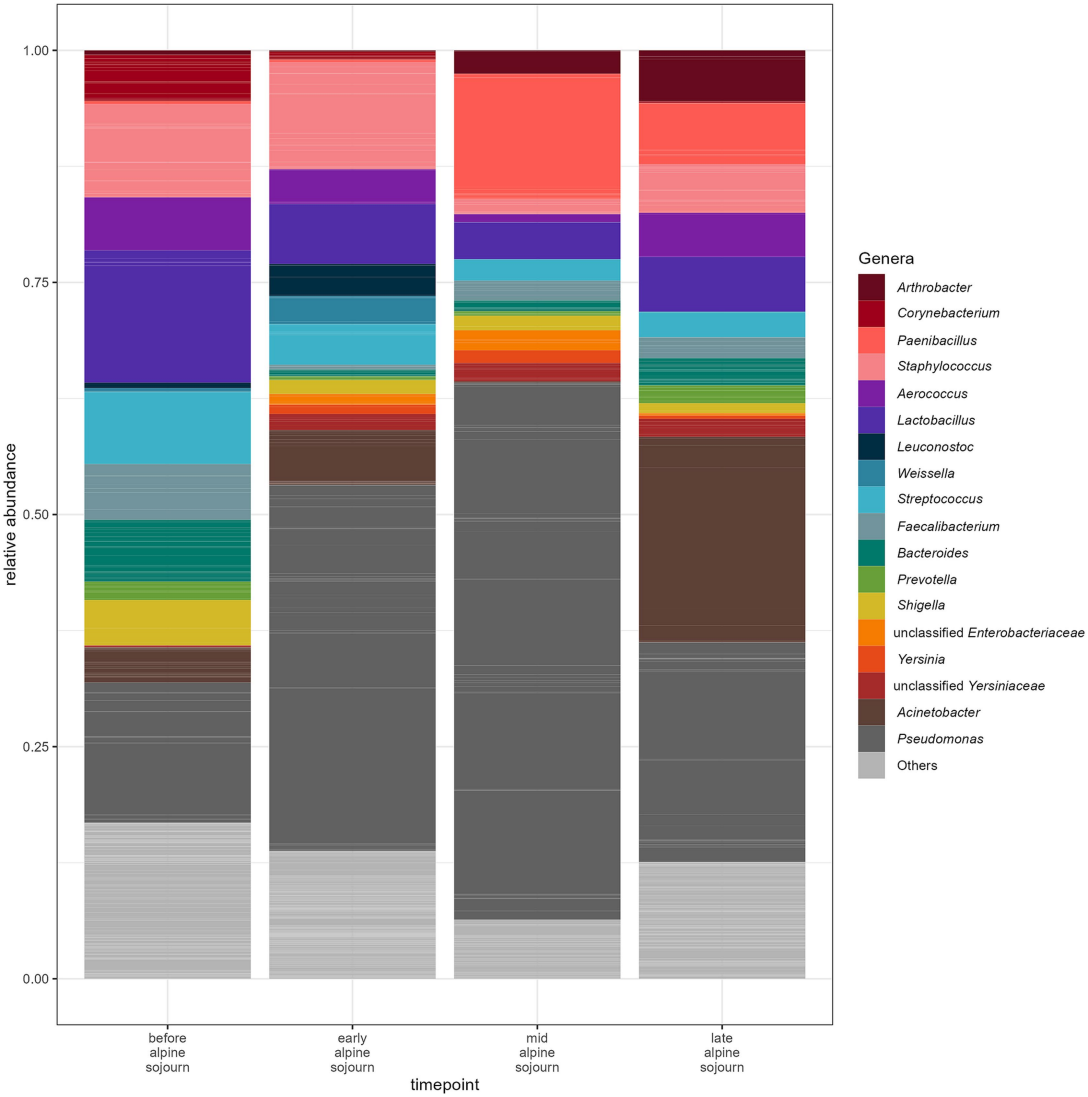
Mean relative abundance of ASVs of milk samples at different times during the alpine sojourn. Individual ASVs are separated by grey lines. ASVs assigned to the same genus are filled with the same color. Only genera are shown with a relative abundance of at least 10% in one individual sample.

The number of ASVs detected in milk samples ranged from 24 to 1,329 ASVs (median: 90.5 ASVs). The bacterial genera with the highest relative abundance in milk samples at different time points are shown in Figure 2*. Pseudomonas* was the most abundant genus throughout the sampling period, accounting for 15.01% at P1 (before alpine sojourn), peaking at 39.44% mid-season (P3), and declining to 23.66% at the end of the alpine season (P4). The relative proportion of *Lactobacillus* was 14.23% at P1, but decreased to approximately 6% at subsequent time points. This does not necessarily reflect a decline in absolute abundance, but rather a shift in the overall microbial community structure. *Staphylococcus* was highly abundant (>10%) only at early time points. *Paenibacillus* remained at low relative abundance during P1 and P2 (~0.3%) but increased to 13.50% at P3 and 6.59% at P4. *Acinetobacter* showed fluctuating relative proportions: 3.80% at P1, 5.92% at P2, a drop to 0.19% at P3, and a notable increase to 22.13% by the end of the alpine season.

**Figure 3:**
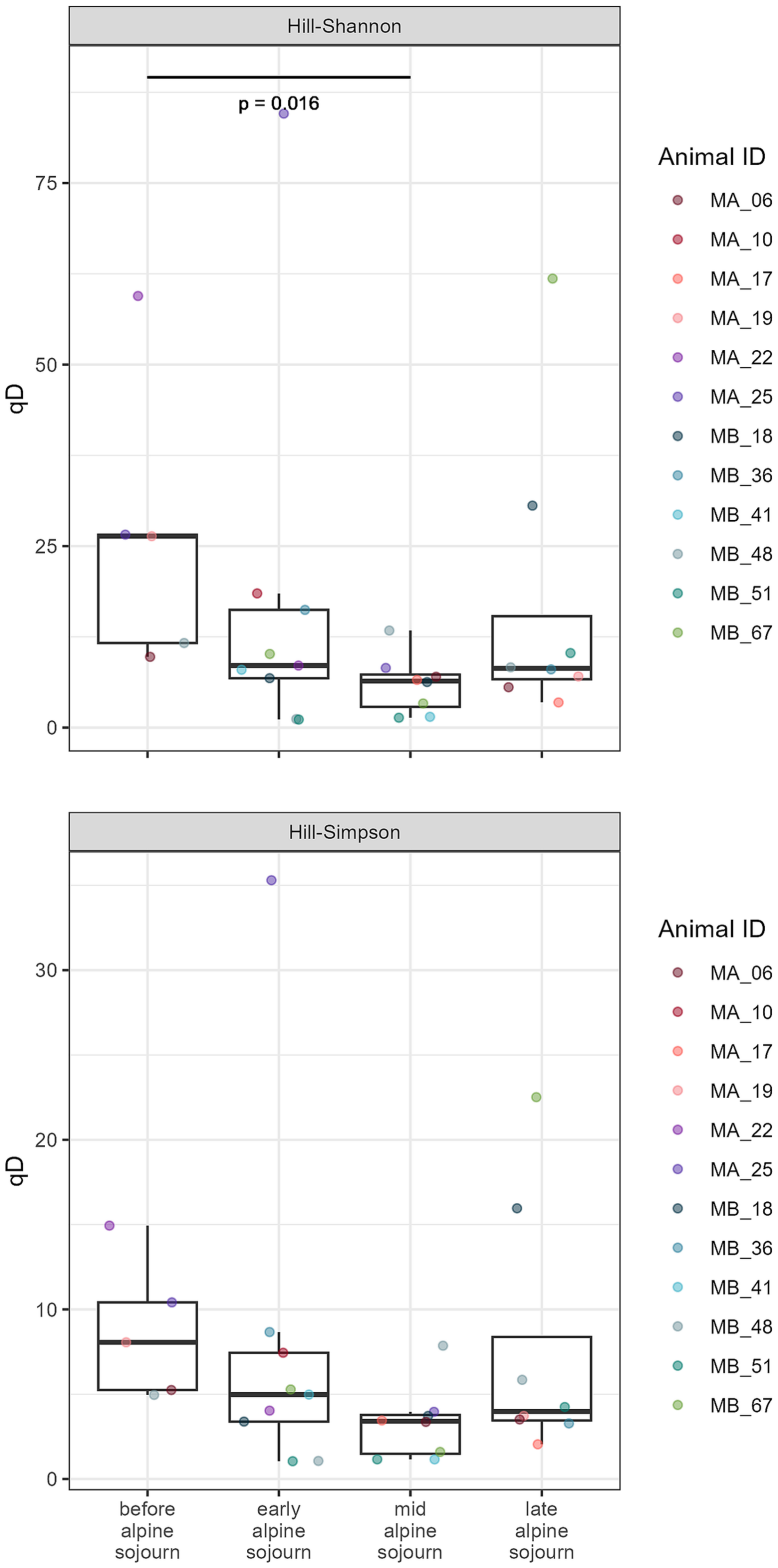
Boxplots comparing Hill-Shannon and Hill-Simpson indices in milk samples at different timepoints during alpine sojourn. The Box represents the interquartile range (IQR) between 0.25 and 0.75, the horizontal line represents the median and the whiskers ranges to the last data points within the 1.5 times IQR. Additionally, the individual data points are displayed in color according to animal ID.

Significant differences in alpha diversity were found before and during the alpine sojourn until the mid-alpine sojourn period in milk samples (Figure 3). Overall, the Kruskal-Wallis test identified statistically significant differences between the groups (Chi2 = 8.951, *p* = 0.030) within the Hill-Shannon indices. The post-hoc Dunn’s test with Bonferroni alpha correction revealed statistically significant differences between the time before the alpine sojourn and the mid-alpine sojourn (*p* = 0.016).

**Figure 4:**
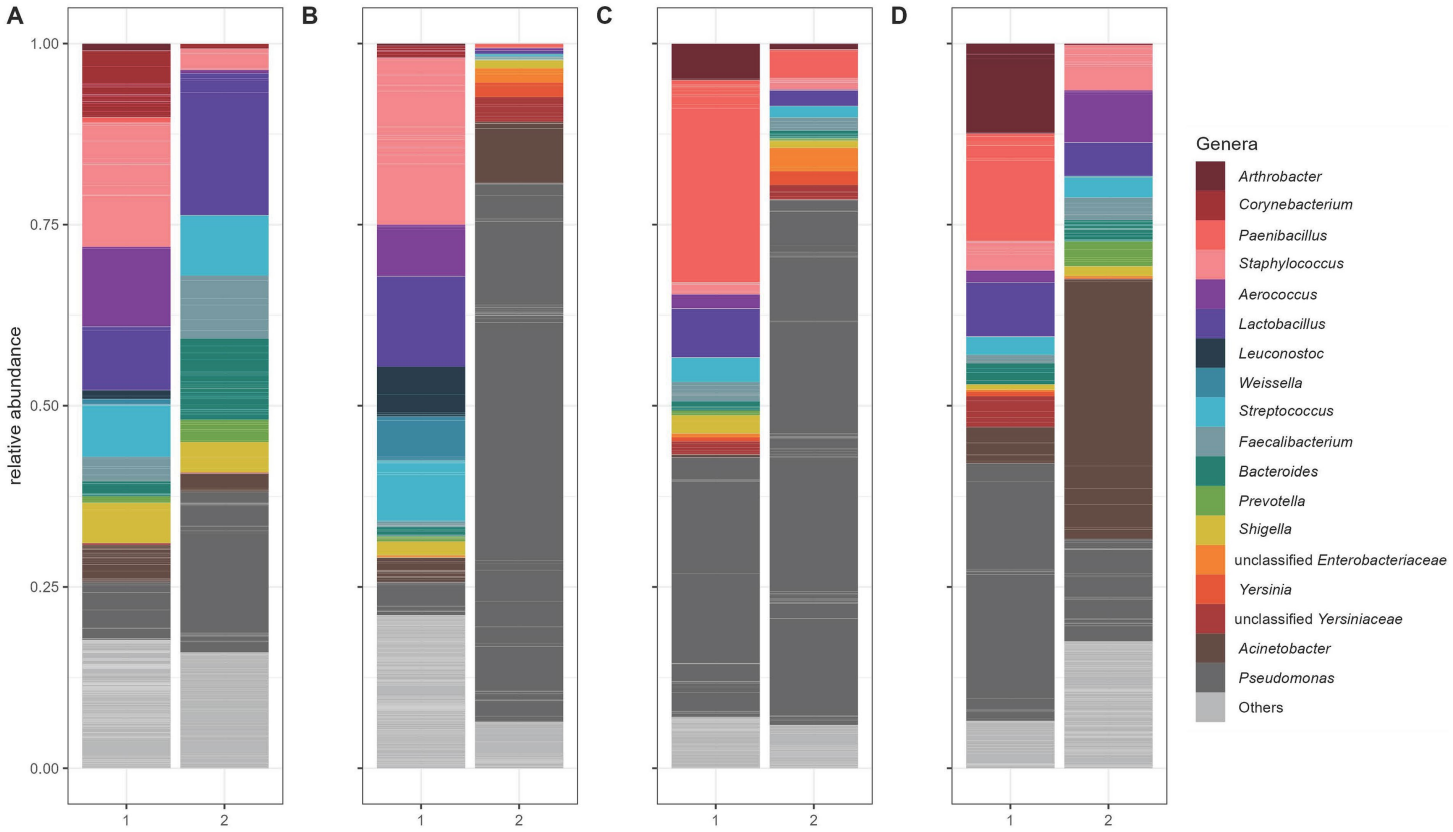
Relative abundance of the 16S rRNA gene ASVs of milk samples at the different time points from farm A (1) and farm B (2): **(A)** before alpine sojourn **(B)** early alpine sojourn **(C)** mid alpine sojourn and **(D)** late alpine sojourn. Individual ASVs are separated by grey lines. ASVs classified in the same genus are filled with the same color. Only genera with a relative frequency of at least 10% in at least one individual sample are presented.

**Figure 5:**
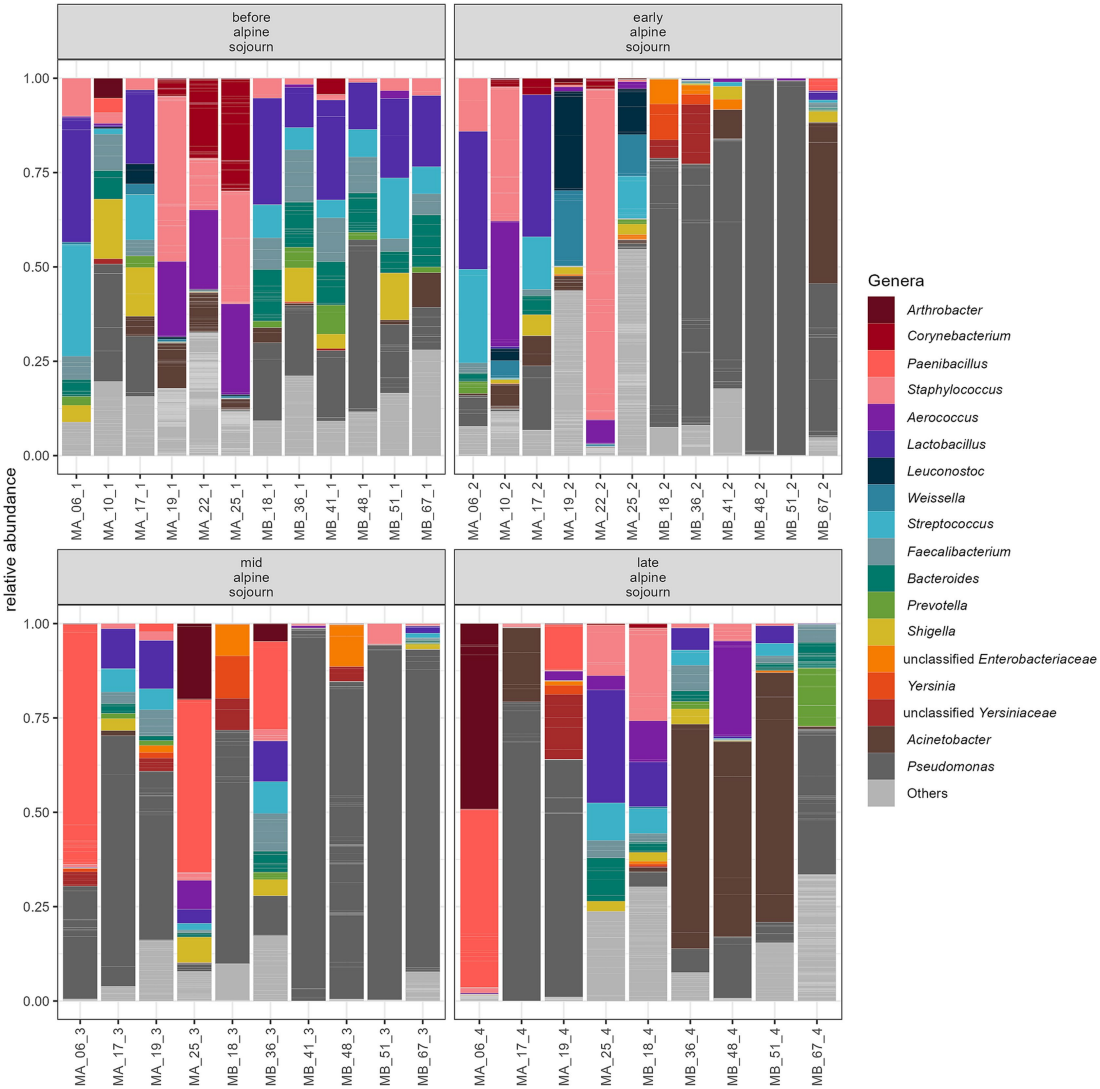
Relative abundance of ASVs at different time points of alpine sojourn. Individual ASVs are separated by grey lines. ASVs classified in the same genus are filled with the same color. Only genera with a relative frequency of at least 10% in at least one individual sample are presented.

Microbial abundance patterns in the raw milk samples at the different sampling points (P1, P2, P3, P4) varied between the two farms (Figure 4). The genus *Lactobacillus* was more evenly distributed in milk samples from farm A, with values ranging from 6.79% at P3 to 12.48% at P2. In farm B at the first sampling time point (P1) *Lactobacillus* spp. was found at higher levels (19.62%), while it had significantly lower relative abundances at other sampling time points (P2 0.43%, P3 2.13%, P4 4.61%). For *Pseudomonas* spp. there were clear differences between farms. On farm A, values for detecting *Pseudomonas* spp. at sampling points P3 and P4 were higher than at the first two sampling time points (for P1 7.99%, P2 4.60% P35.83% and P4 35.56%). On farm B, *Pseudomonas* spp. were found at a higher level with 22.20% at P1, 74.28% at P2 and dominates at P3 with 72.46% but had lower relative abundances at the last sampling point P4 (14.14%). On farm A, *Staphylococcus* spp. were abundant at the first two sampling points (17.17% in P1 and 23.40% in P2). In farm B, however, the values for *Staphylococcus* spp. were overall lower, with the proportion being highest at P4 (6.20%). A particularly high value for *Acinetobacter* spp. was identified in farm B, especially at P4 with 35.85%. In farm A, the values for these species were comparatively lower and range between 0.41 and 5.11%. Further relevant differences existed for *Paenibacillus* spp., which occurs in farm A at P3 at 27.88% and at P4 at 14.83%. These species were hardly detectable in farm *B. bacteroides* showed a higher proportion in farm B at P1 at 11.23%, while the values in farm A were much lower. The results of the milk samples collected from each animal are presented in Figure 5.

### Curd and cheese core

For the curd samples, the highest relative abundance was accounted for *Pseudomonas* with 45.59%, followed by *Acinetobacter* (11.70%), and the *Shigella* complex (8.87%), *Lactobacillus* (5.44%) and *Hafnia-Obesumbacterium* (4.11%). Other genera detected were *Faecalibacterium* (4.76%), *Prevotella* (3.58%), *Bacteroides* (3.29%), *Streptococcus* (2.79%) and *Staphylococcus* (0.36%). Like in milk, there were high differences at the farm level, e.g., *Pseudomonas* reached values of up to 84.49% in samples from farm B compared to 6.69% from farm A. In farm B the proportion of *Acinetobacter* was 14.64% compared to 8.77% in farm A. Despite these very dominant genera in farm B, the overall microbial diversity detected in the curd was similar in both farms (72 and 62 ASVs for farm A and farm B, respectively).

**Figure 6:**
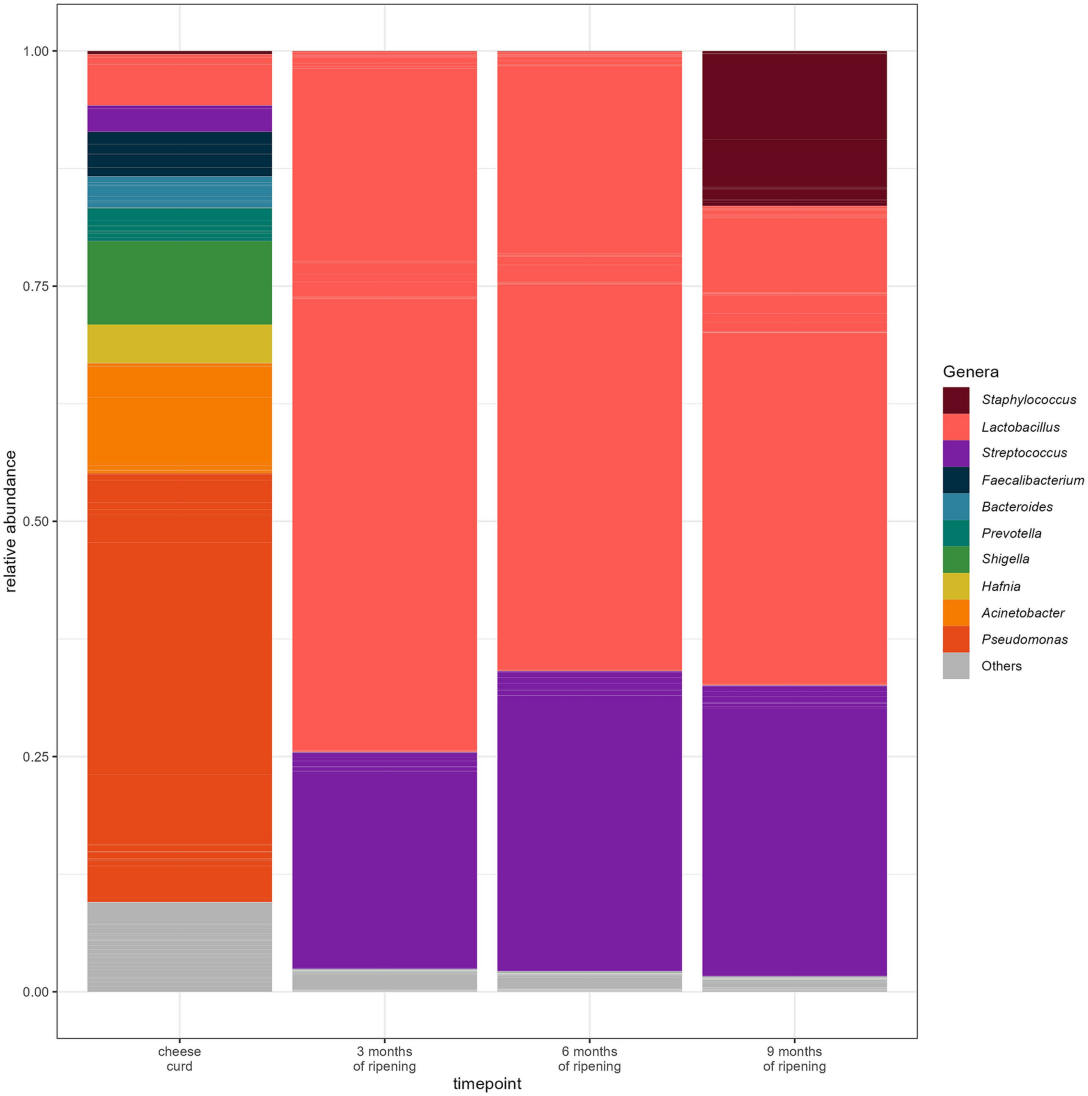
Mean relative abundance of ASVs at the different time points of cheese ripening. Individual ASVs are separated by grey lines. ASVs classified in the same genus are filled with the same color. Only genera with a relative frequency of at least 10% in at least one individual sample are presented. Cheese curd consisted of samples R1, R2, and R3.

After 3 months of cheese maturation (R1), *Lactobacillus* was the largest genus with 74.50%, followed by *Streptococcus* (23.09%). After 6 months of ripening (R2), the relative abundance of *Lactobacillus* was 65.89% and *Streptococcus* 31.92%. At the last sampling time point of 9 months (R3), *Lactobacillus* reached 50.97%, the *Streptococcus* was detected at 30.95% and *Staphylococcus* at 16.47% (Figure 6).

**Figure 7:**
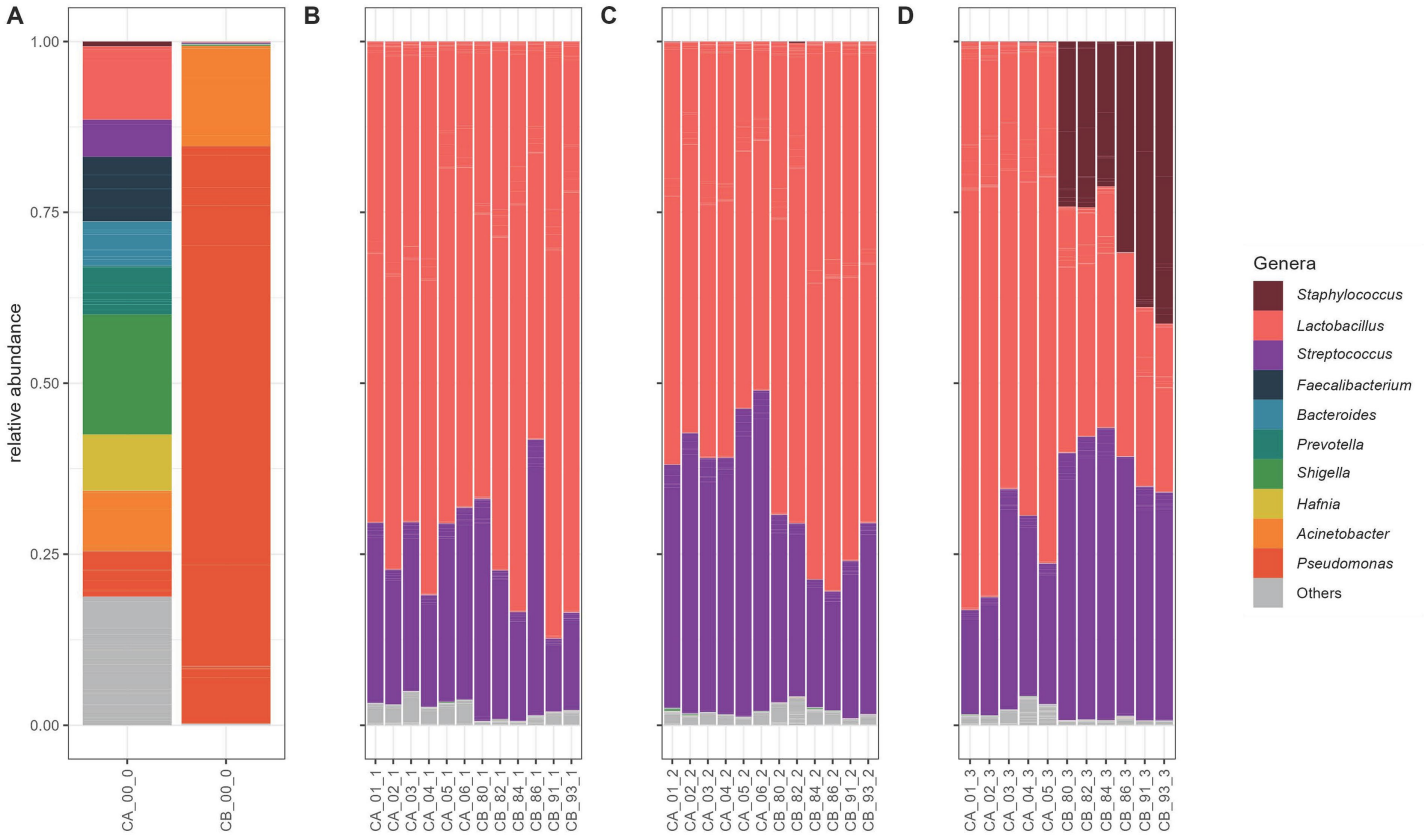
Relative abundance of ASVs of the cheese curd and core samples at the different time points of cheese ripening. Individual ASVs are separated by grey lines. ASVs classified in the same genus are filled with the same color. Only genera with a minimum of 5% relative abundance in at least one of the individual samples are presented. Core samples belonging to farm A (CA) and farm B (CB). **(A)** Cheese curd, **(B)** 3 months of ripening, **(C)** 6 months of ripening, and **(D)** 9 months of ripening.

The genus *Lactobacillus* was the largest group found in all cheese core samples at all three-sampling time points. The genus *Streptococcus* was more abundant in samples of farm A at the second sampling time point (R2) compared to farm B but shifted at the last sampling point (R3) to farm B. The genus *Staphylococcus* was more abundant in the samples from farm B compared than Farm A (Figure 7).

PERMANOVA revealed significant differences between R1 and R3 (*p* < 0.001), as well as between R2 and R3 (*p* < 0.001), but not between R1 and R2 (*p* = 0.602). There were also significant differences between farm A and farm B (*p* < 0.001). However, tests for homogeneity of group dispersions using betadisper were significant for both timepoint and farm comparisons (*F* = 26.98, *p* < 0.001; *F* = 12.59, *p* = 0.001, respectively), indicating that differences in within-group dispersion may contribute to the observed patterns. Therefore, PERMANOVA results should be interpreted with caution.

**Figure 8:**
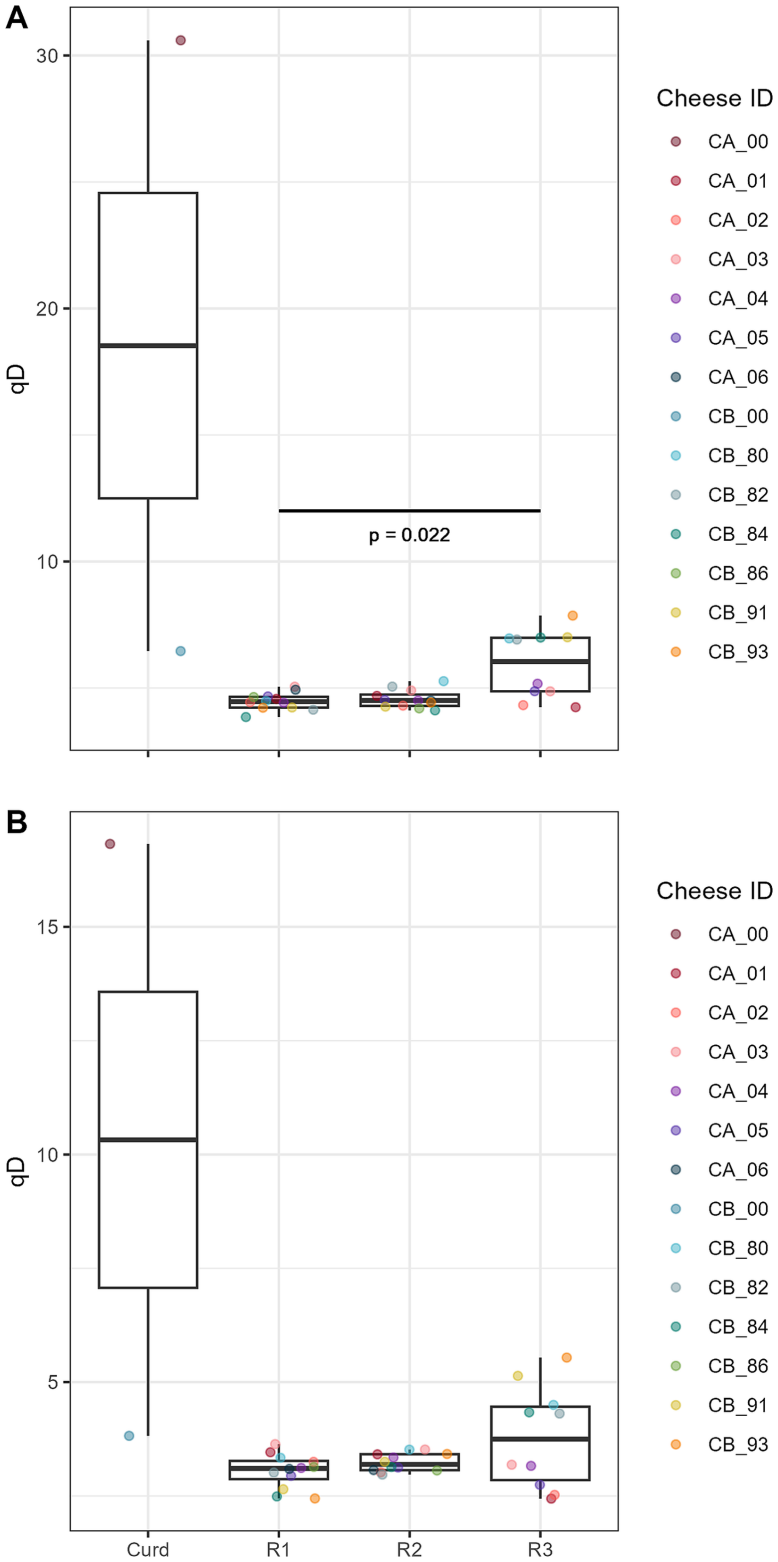
Boxplot comparing Hill-Shannon **(A)** and Hill-Simpson **(B)** indices of time points during cheese ripening. The Box represents the interquartile range (IQR) between 0.25 and 0.75, the horizontal line represents the median and the whiskers ranges to the last data points within the 1.5 times IQR.

The bacterial alpha diversity in the cheese core samples and in the samples at different stages of ripening of VA was calculated using Hill-Shannon and Hill-Simpson diversities (Figure 8). Overall, the Kruskal-Wallis test detected significant differences between the groups (Chi2 = 12.08, *p* = 0.007) within the Hill-Shannon indices. *Post hoc* Dunn’s test with Bonferroni correction identified a significant pairwise difference between 3 months and 9 months of ripening (*p* = 0.022).

## Discussion

This study demonstrates that despite dynamic changes in the milk microbiota during alpine pasturing, the clinical health status of dairy cows remained largely stable. Across the three examined time points, the incidence of clinical abnormalities decreased at farm A and stabilized at farm B. Most clinical findings were minor, including mild respiratory signs, subclinical mastitis, or superficial skin lesions. Notably, just one case of systemic illness was observed, and most animals maintained physiological body temperatures and exhibited normal behavior throughout the alpine season. This one cow showed signs of ketosis a week after alpine sojourn. A negative energy balance leads to the mobilization of body fat, whereby excess fatty acids are converted into ketone bodies in the liver, which leads to ketosis at elevated concentrations. High-yielding and older animals as well as animals with twin pregnancies are particularly at risk, especially in the period shortly before to 6 weeks after birth. While subclinical ketosis is more common and difficult to recognize, the clinical form manifests itself through symptoms such as variable appetite, hard feces and a typical odor. In severe cases, central nervous disorders and even comatose states can occur (Fürst-Waltl and Schwarzenbacher, 2021).

Overall, our study suggests a potential beneficial effect of pasture farming on the udder health. Factors like somatic cell count, infection status, lactation number, and stage of lactation in the valley affect udder health during alpine summer pasturing (Walkenhorst et al., 2005). In this study fewer cows (P1 = 36 compared to P3 = 30) showed signs of clinical or subclinical mastitis over the pasture period, confirming other studies (Kaczorowski et al., 2022; Steinberg et al., 2022).

Using 16S rRNA gene sequencing, *Pseudomonas* spp. emerged as the dominant genus in milk during mid-season, while the relative abundance of *Lactobacillus* spp. declined. Despite these compositional changes, none of the cows exhibited clinical signs of illness, underscoring that alterations in milk microbiota do not necessarily indicate compromised health. This observation aligns with findings by Albonico et al. (2020) who reported that raw milk microbiota composition is more strongly influenced by herd identity and management practices than by health status per se. Additionally, the moderate increase in alpha diversity (Hill-Shannon index) observed during mid-season, which was statistically significant compared to the pre-alpine period, was not associated with any adverse physiological effects. When compared with the results of Lima et al., who identified strong microbial signatures linked to mastitis, such as increased levels of Enterobacteriaceae and Streptococcaceae (Lima et al., 2018), our cohort’s microbial profile did not reflect a disease-associated microbiome. The detection of species such as *Staphylococcus warneri* or *Streptococcus viridans* in a few milk samples (Supplementary Table 4) appeared isolated and clinically irrelevant. Overall, our findings support the perspective of Carafa et al. (2020), saying that microbial shifts in alpine milk are best interpreted as ecological adaptations influenced by environmental conditions, production technologies, and farm practices, rather than as indicators of disease.

Alpine grazing enhances cow welfare by providing nutrient-rich feed, reducing stress, and improving health through natural behavior and a cleaner environment (Charlton and Rutter, 2017). Due to the naturally varying feeding conditions on alpine pastures, the milk does not have standardized properties but fluctuates in its quality, such as protein content and other milk components (Povolo et al., 2013). Secchi et al. (2023) conducted a study that highlighted a differences in the microbiome of cheese produced from milk of cows that had grazed on the alpine pastures compared to those that grazed in lowland farming areas (Secchi et al., 2023). Milk of cows from highland pastures exhibited a relative increase in LAB and probiotic species, accompanied by a reduction in spoilage bacteria. This trend was reversed when the cows returned to the valley pastures in fall (Carafa et al., 2020). In our study this effect was not observed. This discrepancy could be attributed to the high inter-alpine variability in botanical composition, altitude, microclimatic conditions, and farm-specific management practices, all of which can profoundly influence the microbial ecology of raw milk and cheese.

In this study, farm-specific variation in BCEs in the raw cow milk used for Alpkäse cheese production was detected. Microbial transfer to milk may be influenced by environmental conditions that suppress microbial populations, dietary shifts, particularly in botanical diversity, and differences in the microbiological environment of alpine pastures (Quijada et al., 2020). During cheese ripening, we did not observe significant changes in the abundance of BCE in the core of VA, indicating a relatively stable bacterial BCE over time. To our knowledge, this is the first qPCR-based dataset quantifying bacterial DNA in VA milk, curd, and cheese core samples. While our results focus on the cheese core, they show a similar overall trend to findings from our previous study on Vorarlberger Bergkäse (VB), which analyzed bacterial communities in the cheese rind at multiple time points, including 0, 14, 30, 90, and 160 days of ripening (Schmitz-Esser et al., 2018). In both cases—Alpkäse core (this study: 3 and 6 months) and VB rind (previous study: 90 and 160 days)—a decreasing trends in BCE per gram was observed over time. However, the reduction was more pronounced in the VB rind than in the Alpkäse core. These differences may reflect the distinct physicochemical and microbial environments of the matrices involved: the cheese core, being more anaerobic and nutrient-dense, likely supports different microbial dynamics compared to the more exposed and oxygen-rich rind. Notably, rind samples were not collected at 9 months, limiting our direct comparison beyond 160 days. Understanding the relationship between the microbial diversity during ripening and the achievement of the expected quality of the product quality is of utmost importance. Most cheeses are made from milk, rennet, microorganisms and salt (Beresford et al., 2001). Microorganisms can either occur naturally or be added artificially (Yeluri Jonnala et al., 2018). In our study, we sampled the curd of cheeses before they were pressed into the mould. Taxonomic profiling revealed that several genera, among them the *Shigella* complex, *Faecalibacterium, Acinetobacter* and *Lactobacillus* dominated in the curd of farm B, whereas *Pseudomonas* dominated in the curd of farm B, followed by *Acinetobacter*. The predominance of *Pseudomonas* prior to moulding poses a risk of spoilage. *Pseudomonas* spp. is commonly associated with contamination in cold environments, such as cold rooms, storage areas, cheese production facilities with a continuous cold chain, and water sources, including cleaning water. *Pseudomonas* spp. will be outcompeted by starters added for cheese production, but may cause mis-odor (Fusco et al., 2020). The detection of 72 and 62 ASVs for farm A and farm B, respectively, highlights the diverse microbial community already present in the cheese curd.

It should be noted that bulk milk samples were not collected under sterile conditions to also include the teat microbiota after washing. This approach allowed us to simulate a sampling situation as close to reality as possible, considering that the milking parlor is also not operated under sterile conditions.

The teat surface is colonized by a diverse microbiota, including Enterobacteriaceae, LAB and *Pseudomonas* (Montel et al., 2014). Teats can be cleaned using various methods, as described in the material and methods. These different cleaning methods lead to different teat microbiota, which gets into the raw milk during the milking process.

For the cheese core samples, there are significant differences in beta diversity between farm A and B. However, a significant result from the test for homogeneity of group dispersions indicates that this difference may be influenced not only by differences in microbial community composition but also by differences in within-group variability. This suggests that farm-specific factors, such as environmental conditions, milk microbiota, or production practices, may influence microbial community structure and stability. Over both farms, there is no significant difference between R1 and R2 but between the earlier stages (R1 and R2) and the later stage (R3). This suggests that the microbial communities undergo substantial changes as the cheese ripens, and that the microbial composition remains relatively stable between the two earlier ripening stages. This is in accordance with a recent study that examined differences in the microbial transcriptome after 30 and 90 days of ripening in VB (Quijada et al., 2022).

The different microbial diversity observed in the cheese curd from the two farms likely reflects differences in management practices, such as variations in raw milk storage conditions, teat cleaning protocols, and milking system configurations. Our results align with previous studies (Lecaudé et al., 2024; Montel et al., 2014), which demonstrate that farm-specific practices can impact microbial succession and functional properties during cheese production.

The surface ripening process of VA plays a crucial role in shaping its sensory and physicochemical properties. Although this study focused on the microbial dynamics of the cheese core, the rind microbiota is equally important, as it contributes to the development of flavor (Quijada et al., 2022). Surface-ripened, brine-washed cheeses like VB, which are produced using similar procedure, are typically colonized by a diverse range of bacteria and fungi, including *Brevibacterium*, *Corynebacterium*, and *Penicillium*, which are influenced by environmental conditions and production practices (Quijada et al., 2020). Differences in salting methods, rind washing frequency, and ripening environments between farm A and farm B may lead to variations in the rind microbiota, so future studies should also investigate the microbial communities on the rind of VA.

The microbiota of raw milk is highly complex, comprising a wide variety of bacteria, with *Lactobacillus* spp. only accounting for only a small proportion (Bottari et al., 2018). In our study, *Lactobacillus* spp. ranged between 3.99 and 14.22%. *Lacticaseibacillus*, a genus that was split from the larger *Lactobacillus* genus during the reclassification by Zheng et al. (2020), was ranked as 16^th^ most abundant genus in our study. *Lacticaseibacillus* are particularly known for their ability to produce aromatic compounds (e.g., diacetyl, acetoin) that enhance the sensory profile of cheese. *Lacticaseibacillus* species are often active during cheese ripening, where they contribute to the breakdown of peptides and amino acids, also leading to the development of complex flavors. In our study, *Lacticaseibacillus* had similar relative abundances throughout ripening (after 3 months: 0.02%, after 6 months: 0.02%, after 9 months: 0.01%) and had a best BLAST hit (NCBI) with a *Lacticaseibacillus paracasei* strain (100% nucleotide identity, GenBank: MW692396.1), which is assumed to have an important role in flavour development (Bettera et al., 2022; Parente et al., 2020). In total 12 highly similar *Lacticaseibacillus-* associated ASVs were detected in this study, from which 6 were also detected in some of the milk samples.

The use of starter cultures is an effective approach to improving the hygiene and safety of raw milk cheese without compromising its traditional microbial diversity. Lucchini et al., 2018 showed that Natural Milk Starter Cultures (NMC) consisting of *Streptococcus thermophilus* and *Lactobacillus delbrueckii* subsp. *bulgaricus*, led to a faster and more stable pH reduction, which inhibited the growth of undesirable microorganisms. Importantly the microbial diversity was maintained in all farms, regardless of the use of NMC. This approach can help to reduce pathogenic bacteria and ensure a more stable cheese fermentation, particularly in alpine dairies where hygiene conditions may be sub-optimal (Lucchini et al., 2018)*. Lactobacillus* spp. and *Streptococcus* spp. are among the starter cultures commonly used in traditional cheese making processes (see Figure 1) (Montel et al., 2014). During ripening, the genus *Lactobacillus* dominated the microbial community at the first sampling time point (R1), 3 months after cheese production. By 9 months of ripening, a marked increase in *Staphylococcus* abundance was observed, coinciding with a 15% decrease in *Lactobacillus*. This shift suggests a succession of the microbial community, likely driven by changes in the cheese matrix—such as reduced moisture, increased salt concentration, or nutrient depletion—which favors the growth of salt-tolerant and desiccation-resistant genera like *Staphylococcus*. In contrast, the relative abundance of *Streptococcus* remained stable throughout the entire ripening period. This indicates that it either maintained a competitive niche or was metabolically less sensitive to changes in the ripening environment. Its persistence might reflect a commensal role or a tolerance to the evolving physicochemical conditions. During the ripening process, *Staphylococcus* and *Lactobacillus* clearly dominated the cheese core, being in accordance with literature (Carafa et al., 2019).

After 9 months of maturation (R3), the cheese core showed the greatest difference between farm A and farm B. In cheese from farm B, the genus *Staphylococcus* accounted for about a third of the microbiota, whereas it represented only a relative abundance of less than 1% in farm A. The high relative abundance of *Staphylococcus* in cheese from farm B could be indicative of differences in hygiene practices during milking, raw milk handling, or cheese production. *Staphylococcus* species, including both coagulase-positive and coagulase-negative strains, are commonly associated with the skin and mucosal surfaces of animals and humans, as well as with environmental contamination in dairy facilities (Irlinger, 2008; Natsis and Cohen, 2018; Verdier-Metz et al., 2012). Poor sanitation of equipment, suboptimal cleaning of teats, or contamination during cheese handling could lead to the introduction and persistence of *Staphylococcus* in the cheese microbiota (Verdier-Metz et al., 2012). While *Staphylococcus* is often associated with contamination, certain coagulase-negative *Staphylococcus* species (e.g., *Staphylococcus xylosus* and *Staphylococcus equorum*) are known to play a role in cheese ripening (Schornsteiner et al., 2014). These species can contribute to flavor development through proteolysis and lipolysis (Quijada et al., 2022). Further analysis, such as species-level identification and toxin profiling, would be necessary to determine whether the *Staphylococcus* population in farm B cheese is beneficial or problematic.

During ripening, thermophilic LAB from cultures are dominant in the core at all ripening stages and consist mainly of *Lactobacillus delbrueckii*, *Streptococcus thermophilus*, *Lactobacillus helveticus* and *Limosilactobacillus fermentum* (*formerly* nomenclature: *Lactobacillus fermentum*) (Gatti et al., 2008; Zheng et al., 2020). This observation aligns with our findings, as the starter cultures used by the farms were *Streptococcus thermophilus* and *Lactobacillus delbrueckii*. Studies have shown that *Lactococcus lactis*, another starter culture, can persist throughout the ripening process, potentially entering a viable but non-culturable state while maintaining metabolic activity (Ruggirello et al., 2016). The *Lacticaseibacillus casei* group, initially present in low numbers in raw milk, becomes dominant during ripening and plays a crucial role in flavor development (Bottari et al., 2018). On the other hand, the microbiota composition of farm bulk milk and dairy silo milk significantly influences the microbiota composition and development of a traditional long-ripened cheeses, such as those produced in Sweden (Sun et al., 2023). Psychrotrophic bacteria, which are common in raw milk, disappear as soon as the curd reaches 47–48°C and are therefore not detected in the cheese core like the strictly mesophilic bacteria (Carafa et al., 2020; Giannino et al., 2009). These temperature-depended microbial dynamics are critical for ensuring the safety and quality of the final products.

In conclusion, our research on the microbial dynamics in the ripening process of VA has demonstrated that different manufacturing process conditions significantly affect the microbiological properties of the cheese core. By selecting farms with distinct manufacturing practices, we were able to track and analyse the microbial composition of locally produced cheeses, focusing specifically on the core during the ripening of VA cheese.

Our findings indicate that the microbiota of the cheese core is primarily shaped by the production conditions prevalent on the alpine pastures. Notably, we observed that the microbiota in VA cheese curd is significantly more differentiated prior to pressing. As the cheese ripens, the microbial community within the core rapidly organizes itself, leading to the establishment of a stable and consistent cheese microbiota. We hypothesize that the stability is crucial for developing the desired sensory and physicochemical properties of the cheese. Understanding these farm-specific variations not only provides insights into the microbial dynamics of VA but also emphasizes the importance of production conditions in shaping the unique characteristics of alpine cheeses. This knowledge can be leveraged to optimize cheese-making practices and improve the quality and safety of traditional dairy products in the Austrian Alpine region.

## Data Availability

The data for this study have been deposited in the European Nucleotide Archive (ENA) at EMBL-EBI under accession number PRJEB88854 (https://www.ebi.ac.uk/ena/browser/view/PRJEB88854). The R codes used for the analysis are available on GitHub at https://github.com/ffroch/VAcheese/.
