# Supplementary material for: Microbial dynamics in the ripening process of Vorarlberger Alpkäse: farm-specific variations at two Austrian alpine farms
Source: Front Microbiol. 2025 Aug 13;16:1617995. doi: 10.3389/fmicb.2025.1617995 (PMC12380632; doi:10.3389/fmicb.2025.1617995)

Supplementary Figure 1. Abundance of bacterial cell equivalents (BCE) in (**A**) milk samples and (**B**) cheese core samples collected at different time points during the alpine sojourn and ripening time at two Alpkäse farms, as determined by qPCR. Milk samples collected at P1: before alpine sojourn, P2: early alpine sojourn, P3: mid alpine sojourn, P4: alte alpine sojourn. Cheese samples collected at R1: 3 months, R2: 6 months, R3: 9 months. Graph shows median and interquantile ranges for the samples from each farm (A, B) and for each analyzed sampling time.

A

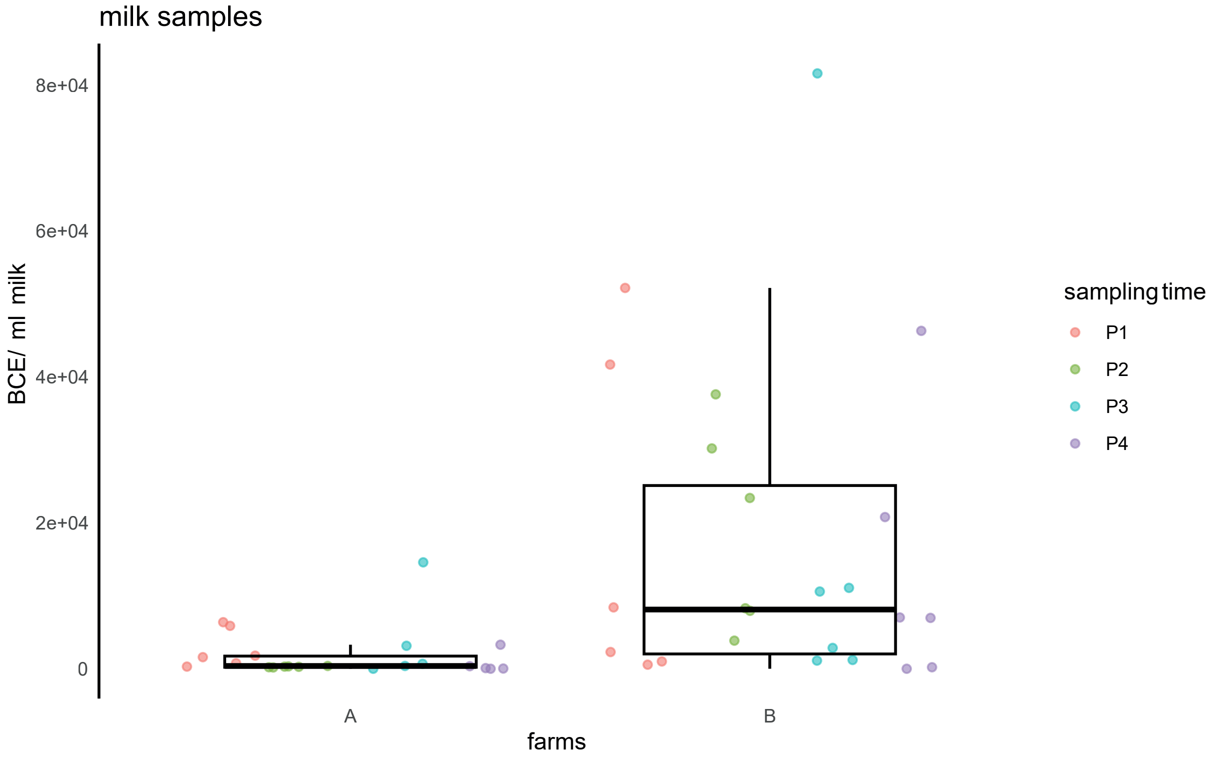

B

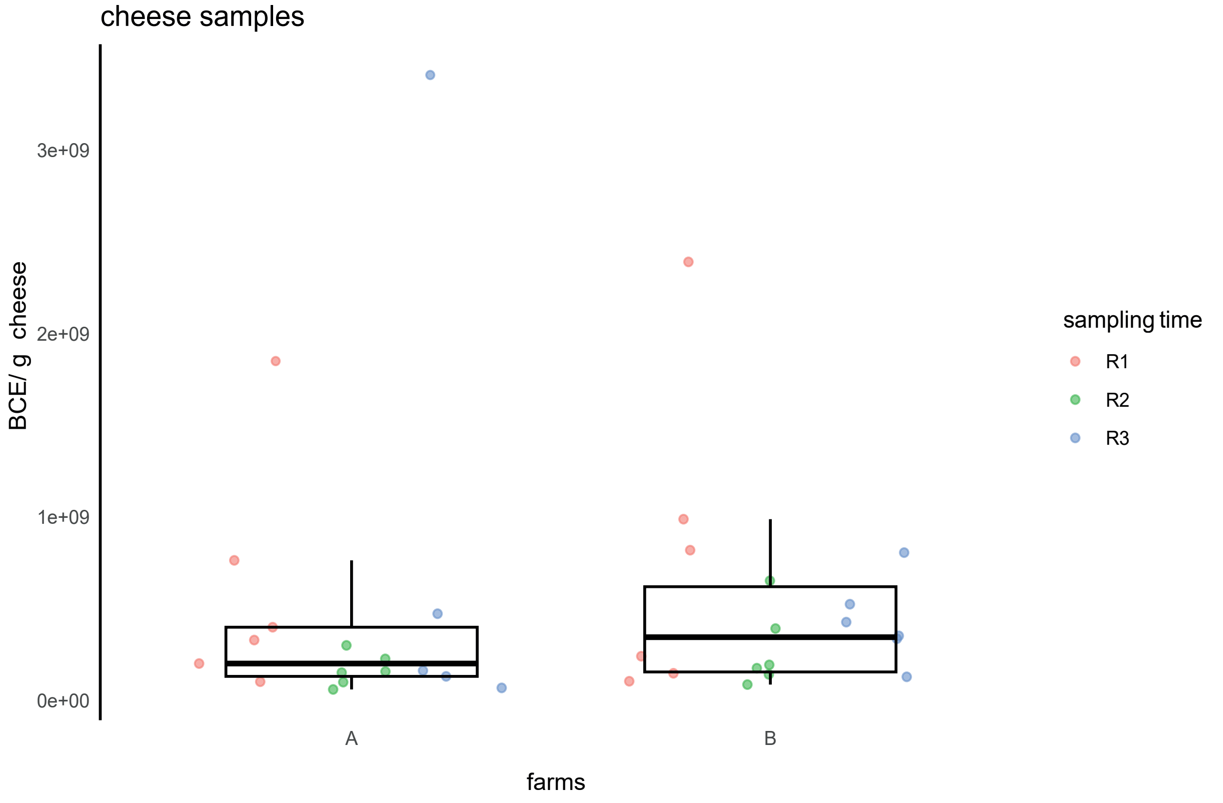

Supplement: Supplementary file 6 [file Data_Sheet_1.pdf]
